# Web malware spread modelling and optimal control strategies

**DOI:** 10.1038/srep42308

**Published:** 2017-02-10

**Authors:** Wanping Liu, Shouming Zhong

**Affiliations:** 1 School of Mathematical Sciences, University of Electronic Science and Technology of China, Chengdu 611731, China; 2 College of Computer Science and Engineering, Chongqing University of Technology, Chongqing 400054, China

## Abstract

The popularity of the Web improves the growth of web threats. Formulating mathematical models for accurate prediction of malicious propagation over networks is of great importance. The aim of this paper is to understand the propagation mechanisms of web malware and the impact of human intervention on the spread of malicious hyperlinks. Considering the characteristics of web malware, a new differential epidemic model which extends the traditional SIR model by adding another delitescent compartment is proposed to address the spreading behavior of malicious links over networks. The spreading threshold of the model system is calculated, and the dynamics of the model is theoretically analyzed. Moreover, the optimal control theory is employed to study malware immunization strategies, aiming to keep the total economic loss of security investment and infection loss as low as possible. The existence and uniqueness of the results concerning the optimality system are confirmed. Finally, numerical simulations show that the spread of malware links can be controlled effectively with proper control strategy of specific parameter choice.

Secure networks are known to be crucial to cyber business and online payment. However, real computer systems always suffer from malware programs that perform malicious or unwanted operations. With the rapid development of information technologies, the diversity of malicious software evolves largely in the past decades, from traditional computer viruses to current families of mobile viruses, Internet worms, Trojans, Adware, Spyware and so on^1^. Essentially, they can range from being simple annoyances (pop-up advertising) to causing serious malicious invasion, e.g., stealing passwords and valuable data or controlling compromised devices over networks^2^.

Nowadays, the World Wide Web (WWW) is widely and consistently used in business activities, online banking, and e-commerce as well as everyday lives of human beings worldwide. There are over 1 billion websites worldwide, and the number of global Internet users has exceeded 3 billions, according to the online statistical estimates by an International website^3^. But, it is relatively unprotected, and the number of web threats significantly grows as a result of the popularity of the Web. Especially, the appeal of Web 2.0 applications will bring users benefits of greater interactivity and more dynamic websites, but it also further increases the vulnerability of the Web, e.g., suffering greater security risks inherent in browser client processing.

Most of cyber-criminals are now financially motivated to develop new types of malware. Recently, *web-based malware* has seen tremendous growth due to the widespread adoption of mobile devices. Unlike Internet worms^4^,^5^,^6^ that can automatically replicate themselves, web malware usually attack hosts by taking advantage of the vulnerabilities of web pages, and proliferate by means of social engineering. So, user intervention characterizes the spreading process of this kind of malware (e.g., bundled viruses). Hosts infected by web malware can suffer from modifications of browser settings (e.g., default homepage, search bars, toolbars), cause user registry modification, display intermittent advertising pop-ups or even transmit information about your web-browsing habits to advertisers or other third party interests without your awareness. Nowadays, web-based viruses have become an increasingly attractive option for cyber-criminals to attack users without searching for new vulnerabilities in network services. They can spread in the form of hyperlinks (i.e., the addresses of corresponding harmful websites purporting to proliferate malware) which may exist in short messages or spam emails that lure victims to click on the malicious URLs and then redirect to a false web page which is able to inject malware into their devices. In the past few years, the number of browser-based infections has grown exponentially, and malicious links have become a major threat. Thus, there is a need to carefully characterize the spread of web viruses and develop efficient strategies for web malware containment.

Attackers often use social networks to distribute malware^7^,^8^. Researchers of BitDefender claimed that malware originating from harmful links on Facebook was the top attack vector for mobile devices. Spam links on social networks are infecting mobile devices easily since they are often platform-independent. Moreover, financial purposes enormously motivate cyber-attackers to use websites to conduct phishing attacks that attempt to acquire personal or financial information such as usernames, passwords, and credit card details. For instance, some spoofed websites or links are intentionally designed to seem official, or even these sites are legitimate, but have been compromised by malware, SQL injection or other malicious techniques. Typically, phishing is carried out by the way that the user views a phishing message, in spoofing emails or instant messages, and is tricked into clicking a link that leads to a malicious website^9^. Consequently, it is important to make a trial on better understanding of the diffusion of malicious URLs for improving the safety and reliability of devices and networks.

In the past decades, a variety of epidemic models were developed to address the diffusion of disease infections^10^,^11^,^12^ and population dynamics^13^,^14^,^15^,^16^. Especially, spatial effects on herbivore populations are recently studied in structured populations in ecosystems^17^,^18^,^19^. Inspired by the research of biological epidemics^20^, *malware epidemiology* similarly aims to study the dynamics of malware spread over time and analyze the factors affecting its propagation process^21^,^22^. Much effort has also been done in the area of developing mathematical models for malware spread^23^, and most existing models for malicious code are based on deterministic epidemic models^24^,^25^. For instance, some earlier mathematical models were obtained by the compartmental approach, such as epidemic SIS, SIR and SIRS models^26^,^27^, which differ by considering whether the acquired immunity is permanent or not. Modification of theses models generated guides for infection prevention by using the concept of epidemiological threshold^28^,^29^. Some dynamical models were further proposed to give estimations for temporal evolutions of infected nodes depending on network parameters considering topological aspects of the network. But, in most of previous works, susceptible computers were assumed to be instantaneously infective as soon as they were infected and later recovered with a permanent or temporary acquired immunity. In fact, however, a device receiving malware messages will not immediately become infective until the user activates it by clicking on the hyperlink address and successfully accessing the malware websites. On the other hand, in spite of much work having been devoted in the past decades to understanding the spreading behavior of malware^30^,^31^, those models were actually limited to model the propagation of computer viruses and Internet worms. As far as we are concerned, few work focuses on addressing the characteristics of web malware and their propagation dynamics. Besides, empirical results indicate that human dynamics have effects on web malware diffusion. However, little is known about how human behaviors have influences on web malware outbreak and propagation when user’s security awareness is considered. Therefore, this paper aims to establish an elementary dynamical model (relatively simple in the form of ordinary differential equations) to address how web malware spread with the impact of users’ security awareness, and develop proper prevention strategies with human interventions by the optimal control theory.

## Results

### A compartment-based model

#### Web malware and propagation mechanisms

Generally speaking, web malware is a specific kind of malicious programs that use web pages to implement destructions. They usually employ the vulnerabilities of browsers to achieve viral implants by using some malicious codes written in Script. There are different variants of web malware that infect websites, such as iframe viruses. Most of them use iframe HTML code to cause damage by injecting iframe tags into the website^32^. Web threats are able to cause a broad range of risks, such as financial damages, damage of company reputation, and loss of consumer confidence in e-commerce and online banking^33^. Furthermore, multiple types of web malware benefit cybercriminals by stealing confidential information for subsequent sale and help absorb infected devices into botnets.

**Figure 1:**
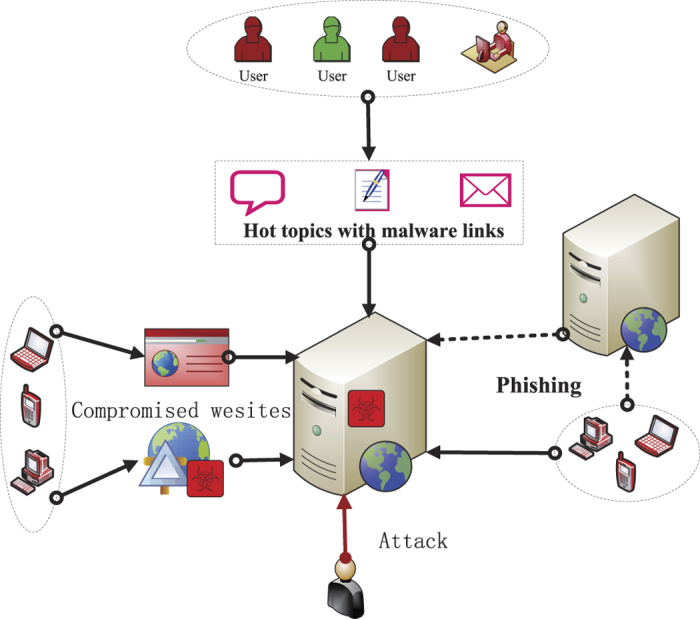
Diagram of web malware spread mechanism. The clients or terminals will get infected once they visit the compromised webpages on the web server which has been intruded.

Attackers exploit the vulnerabilities of browsers or webpages to design and proliferate malicious viruses. Distribution of malicious programs has been largely expanded beyond traditional channels like email viruses to harder-to-avoid approaches like automated “drive-by downloads” launched by infected webpages (see Fig. 1). There are mainly the following several ways for the spread of web threats over networks.

##### Taking fraudulent methods

In this way, phishing and spam are taken to lure users to malicious (often spoofed) websites which can collect information by injecting malware. Network attackers use phishing, DNS poisoning or other means to make them appear to originate from a trusted source^34^.

##### Using social engineering

One fundamental method is to write and forward tempting messages or emails containing the addresses of infected websites. More specifically, malware developers employ social engineering such as enticing subject lines that reference popular personalities, sports, pornography, world events and other hot topics to design malicious links. Once users receive these types of deceptive information and are enticed to click on the hyperlinks which direct to the malicious websites, web viruses will be automatically downloaded and activated, resulting in personal information leak, such as accounts and passwords.

##### Infecting legitimate websites

By this way, legitimate websites infected by web malware will unknowingly transmit malware threats to visitors or alter search results to take users to malicious websites. Upon loading the page, the user’s browser passively runs a malware downloader in a hidden HTML frame without any user interaction.

#### Compartments and parameters

**Figure 2:**
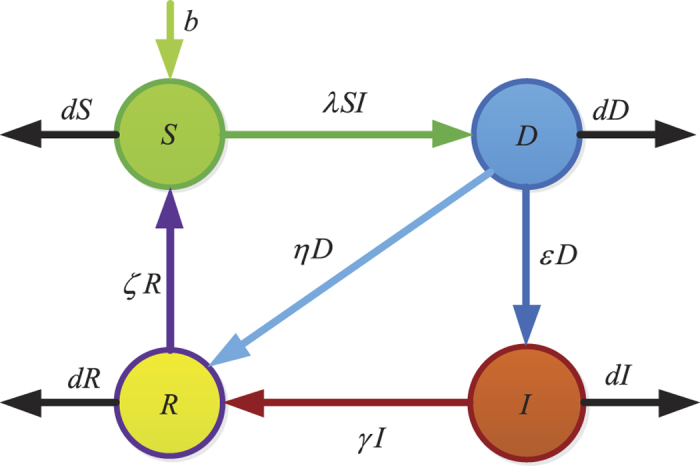
Transition diagram for the SDIRS model. The green (respectively, blue, red, yellow) circle represents susceptible (respectively, delitescent, infected, recovered) nodes (marked by *S, D, I, R*, respectively).

In this section, we aim to develop a new compartmental model to characterize the propagation of web-based malware. For convenience, the devices through which malware propagates are also called as nodes in the sequel. In our model, a host under consideration is assumed to be in one of four states: susceptible(S), delitescent(D) (not yet infective), infected(I), or recovered(R). The state of a node is actually changing over time, i.e., switching among the above four states, because of the proliferation of malware links and the defense of antiviruses. A susceptible node first goes through a delitescent period before being infectious, and a typical pathway of malicious link infection is *S* → *D* → *I* → *R* → *S* (see Fig. 2). Next, several assumptions and parameters are introduced. If a user successfully visits the malware website by clicking on the hyperlink within deceptive messages or spam emails, the host will get infected. In the following, an infected host is assumed to be able to forward the malicious messages through users’ contact lists. Susceptible nodes are assumed to immediately become delitescent as soon as they receive messages containing malicious URLs. Note that user behaviors will play a significant role in affecting the proliferation of malicious hyperlinks. Obviously, vigilant users have a high probability to identity and eliminate malware messages, and update recent security patches to fix bugs for system immunization. Based on this consideration, a parameter *η* is introduced to depict the probability of a D-node leaving for the recovered compartment. On the other hand, users without enough security awareness are probably enticed to click on the malicious links and get infected by the malware automatically downloaded from the insecure websites. Hence, a parameter *ε* is introduced to describe the probability of that a D-node leaves the delitescent compartment for the infected compartment. There is also another case that the states of some D-nodes may keep unchanged, because users may neglect the received malware links and do not take any measures to deal with them.

Infected devices by malware intrusion may exhibit certain symptoms, such as strange disruptions, battery draining and performance clogging. Once abnormal behavior is found, users will take security measures to detect and immune their systems. Thus, we introduce a parameter *γ* to describe the probability that an I-node gets immunization and turns to be recovered.

Immunity is observed when anti-malicious software is run after a node gets affected by malware. However, this kind of immunity is usually temporary. Specifically, when a node is recovered from the infected class, it recovers temporarily, acquiring temporary immunity with certain probability. Because of malware evolution or secure update failure, R-nodes will become back susceptible to malicious infections again. Considering this, a parameter *ζ* is introduced to depict the probability of a R-node leaving for the susceptible compartment owning to immunity failure.

Infective devices will send malicious link copies to their neighboring nodes. For different kinds of malware, the rate of infecting susceptible nodes may be distinguished. This is an important concern for establishing an effective model. Here, the infection rate *λ* is defined as the probability that an S-node receives the malicious link sent by a neighboring I-node within a unit time.

#### Model formulation and analysis

As a matter of fact, the number of nodes in each compartment is dynamically changing over time. Thus, four variables *S(t*), *D(t*), *I(t*) and *R(t*) are introduced to describe the numbers of susceptible, delitescent, infected and recovered nodes at time *t*, respectively. The network size at time *t* is denoted by *N(t*), i.e., *N(t*): = *S(t*) + *D(t*) + *I(t*) + *R(t*).

For simplicity, we assume that all newly-connected nodes are susceptible. The parameter *b* denotes the rate of nodes that are newly connected to the network within per unit time, and the parameter *d* is the disconnection probability that a node leaves the network per unit time. By applying the mean-field technique to the above assumptions, a compartmental model can be formulated as


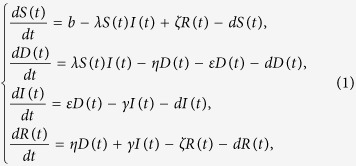


where the parameters *ε, η, γ, b, d, ζ* are nonnegative, and *ε* + *η* < 1.

Adding the equations of system (1) leads to *N*′(*t*) = *b* − *dN(t*), which can be explicitly solved as *N(t*) = *b/d* + (*N*(0) − *b/d)e*^−*dt*^, where *N*(0) represents the initial number of nodes over the network. It can be easily observed that *N(t*) is varying over time if *N*(0) ≠ *b/d*. This corresponds to the fact that real networks are always evolving, owing to certain nodes dynamically connected to or disconnected from the network. While for the special case *N*(0) = *b/d*, the size of the network will keep constant due to a balance of newly-connected and disconnected nodes. The explicit solution also indicates that for the case *N*(0) < *b/d* the total network size *N(t*) will strictly increase to the final saturation number of *b/d*. Actually, the numbers of terminal devices over real networks will also reach saturation by some technological constraints, such as IP addresses, network bandwidth, and communication channel congestions.

**Remark 1**: Note that if *b* = *d* = 0 then system (1) reduces to model web malware propagation over a static network. And, for the case *ζ* = 0 model (1) looks similar to the classical SEIR model with demographics in epidemiology^35^, however, we mainly consider it for the characteristics of web malware propagation and incorporate the impact of human intervention into the model by introducing the appropriate parameter η. Thus, the above SDIRS model is essentially a newly-formulated model for web malware propagation with varying network size.

##### Propagation threshold

The *propagation threshold* of model (1) (usually also called as *basic reproduction number* in epidemic models which can be explained as the average number of secondary infections produced by a single infected node during its infection time) is calculated as (see *Methods A* for detailed calculations)


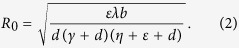


Note that all the parameters in system (1) except for *ζ* have impact on the propagation threshold *R*_0_. This can be explained by that the parameter *ζ* which describes the probability of a R-node losing temporary immunity does not reflect the infective ability of current propagating web malware. By viewing the parameters in (2) as variables, then it obviously follows by the expression of (2) that *R*_0_ is strictly decreasing with respect to the parameters *γ, η, d*, respectively, while *R*_0_ is strictly increasing with respect to another two parameters *b* and *λ*, respectively. For the parameter *ε*, straightforward calculations yield


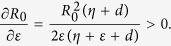


**Figure 3:**
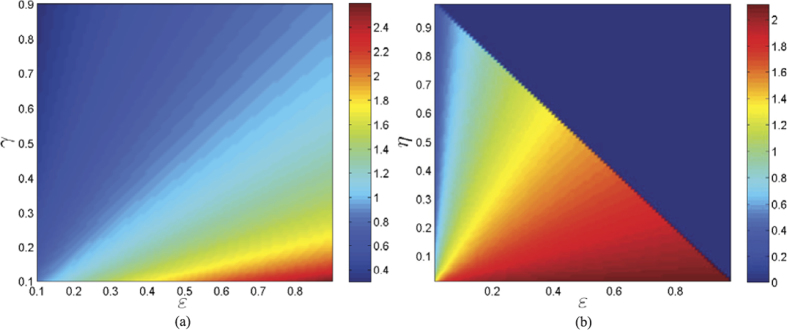
Fixing the parameters *b* = 100, *λ* = 0.00005, *d* = 0.01. (**a**) Values of *R*_0_ as a function of varying *ε* and *γ* with fixed *η* = 0.5. (**b**) Values of *R*_0_ as a function of varying *ε* and *η (ε* + *η* < 1) with constant *γ* = 0.1.

Thus, the threshold *R*_0_ is monotonically increasing with respect to *ε*. The parameters *ε, γ* and *η* are important since they reflect human intervention on malware infection process. Figure 3(a,b) show values of *R*_0_ as a function of two varying parameters *ε* and *γ* (respectively, *ε* and *η*) with other parameters specifically given.

The propagation threshold *R*_0_ plays a significant role in determining the dynamics of system (1). It follows by calculations that system (1) always possesses a malware-free equilibrium point 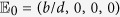 and has a unique malware equilibrium 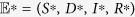 while *R*_0_ > 1, where


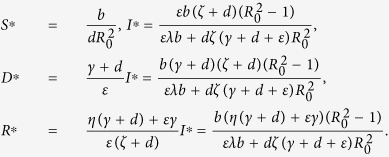


##### Stability analysis

We intend to address the stability of the equilibria of system (1). Firstly, we define the *global stability* of an equilibrium for system (1) with respect to 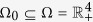. Let 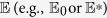 be an equilibrium of system (1), then it is said to be globally asymptotically stable with respect to Ω_0_ if it is *Lyapunov* stable and for each initial value **x**(0) ∈ Ω_0_, then 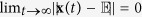, where **x**(*t*) = (*S(t*), *D(t*), *I(t*), *R(t*)).

**Figure 4:**
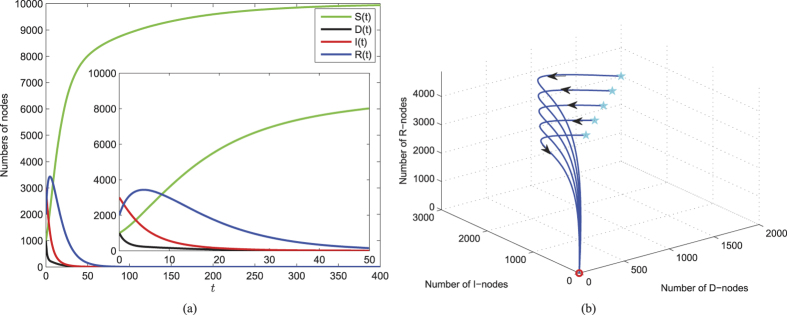
Taking that 100 nodes are connected to the network per unit time, i.e., the connection rate *b* = 100, and nodes are disconnected from the network with the probability *d* = 0.01, and the remaining parameters are chosen as *λ* = 0.00005, *ζ* = 0.1, *η* = 0.5, *ε* = 0.2, *γ* = 0.2. (**a**) Solutions of system (1) with specific initial values *S*_0_ = 1000, *D*_0_ = 1000, *I*_0_ = 3000, *R*_0_ = 2000. (**b**) Phase diagram of *D(t*), *I(t*) and *R(t*) with different sets of initial values.

Then, we theoretically prove the global stability of the malware-free equilibrium 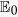 of system (1) with respect to Ω if *R*_0_ < 1 (see *Methods B*). This means that under the model (1) once the threshold *R*_0_ < 1 (under the comprehensive effect of all parameters), then the web malware (for any initial state within Ω) is bound to eventually disappear from the network. In this case, the web malware itself may have low diffusion ability, e.g., the malicious links can be easily recognized, so users will neither click on them nor forward them to other friends. Besides, high security awareness of users also benefits the reduction of *R*_0_ even if the web malware has strong infective ability. Figure 4 numerically illustrates the analytical results. The parameters used for numerical simulations are chosen such that the conditions of the global stability of 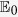 are satisfied. For the set of parameter values given in Fig. 4, the value of *R*_0_ is obtained as 0.8190. Thus, it is apparent from Fig. 4(a) that components of *D(t*), *I(t*), *R(t*) eventually converge to zero, and the component *S(t*) is finally approaching to the saturation number of 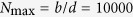. To illustrate the global stability of 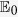, we have plotted the solution trajectories in *D*–*I*–*R* space starting from different initials in Fig. 4(b), in which all trajectories are eventually approaching to the point (0, 0, 0).

For the case *R*_0_ < 1, the global dynamics of (1) in Ω has been completely determined. Its epidemiological implication is that the number of infected nodes over the network vanishes in time so web malware finally disappears from the network. While for *R*_0_ > 1, the web malware will persist. The web malware is said to be endemic if the infected nodes over the network persist above a certain positive level for sufficiently long time. It can be well captured and analyzed through the notion of uniform persistence. System (1) is said to be uniformly persistent (see refs 36 and 37) if there exists a constant 0 < *c* < 1 such that any solution (*S(t*), *D(t*), *I(t*), *R(t*)) with (*S*(0), *D*(0), *I*(0), *R*(0)) ∈ 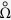 (the interior of Ω) satisfies


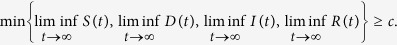


Thus, the web malware is endemic if system (1) is uniformly persistent. And, we can easily prove that system (1) is uniformly persistent by using Theorem 4.3 in ref. 38 (refer to the proof of Proposition 3.3 in ref. 39). In this case, both the numbers of infected and delitescent nodes persist above a certain positive level.

**Figure 5:**
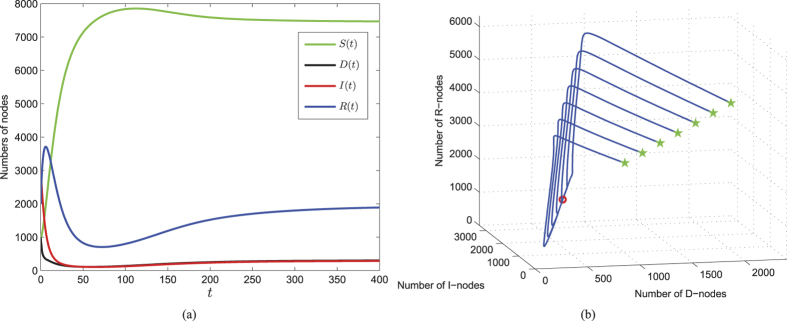
Taking that 100 nodes are connected to the network per unit time, i.e., connection rate *b* = 100, and nodes are disconnected from the network with the probability *d* = 0.01, and the other parameters are chosen as *λ* = 0.0001, *ζ* = 0.1, *η* = 0.5, *ε* = 0.2, *γ* = 0.2. (**a**) Components of system (1) with the specific starting point (1000,1000,3000,2000). (**b**) Phase diagram of *D, I, R* of system (1) with different starting initial points (green stars) within 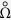.

For the infected equilibrium 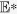 of system (1), we theoretically prove its asymptotical stability if *R*_0_ > 1 and further discuss the global stability of the special case *ζ* = 0 under certain assumptions (see *Methods C*). This means that under the effects of parameters in model (1), once the threshold *R*_0_ > 1, then the number of nodes infected by web malware will finally keep a steady level. This case reflects the kind of web malware which may evolve or have strong infectivity, and thus there exists a game between web malware and antivirus software. Figure 5 numerically illustrate the stability of 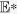. For the set of parameter values specifically given in Fig. 5, the value of *R*_0_ is computed as 1.1582 > 1, and the corresponding infected equilibrium is 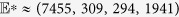. Figure 5(a) shows the evolutions of system (1) with a specific set of initial values, from which it can be seen that all the components of system (1) eventually converge to corresponding infected equilibrium states, respectively. In order to explore how the solutions evolve with different starting points, Fig. 5(b) shows the plot of solution trajectories in *D*–*I*–*R* space starting from different initials. It can be observed that all the trajectories are eventually approaching to the point (*D*^∗^, *I*^∗^, *R*^∗^) = (309, 294, 1941).

##### Parameter analysis

**Figure 6:**
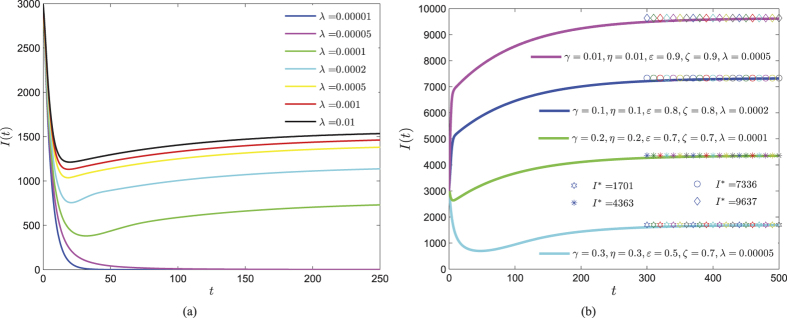
Evolutions of the infected component of system (1) with *b* = 100, *d* = 0.01 and initial vector (1000, 1000, 3000, 2000). (**a**) Taking *ζ* = 0.1, *η* = 0.35, *ε* = 0.25, *γ* = 0.2 and the infection rate *λ* is varying. (**b**) Evolutions of *I(t*) with several different sets of parameters.

For the case *R*_0_ > 1, let 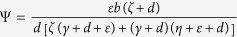, then 
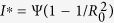
. In Fig. 6(a), it is obviously shown that greater infection rate *λ* benefits the propagation of web malware, resulting in keeping a final higher number of infected devices. It can be also seen in Fig. 6(a) that the infected component of malware equilibrium possesses significant difference when *λ* ∈ [0.00005, 0.001], while *I*^∗^ has inconspicuous increase while *λ* belongs to the interval (0.001, 0.01). By taking several different sets of parameters, the evolutions of *I(t*) are also shown in Fig. 6(b), which indicates that some web malware (characterized by choosing appropriate parameters) is possible to intrude the whole network.

By viewing the parameter *ζ* as a variable, straightforward calculations yield


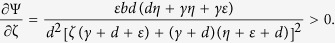


**Figure 7:**
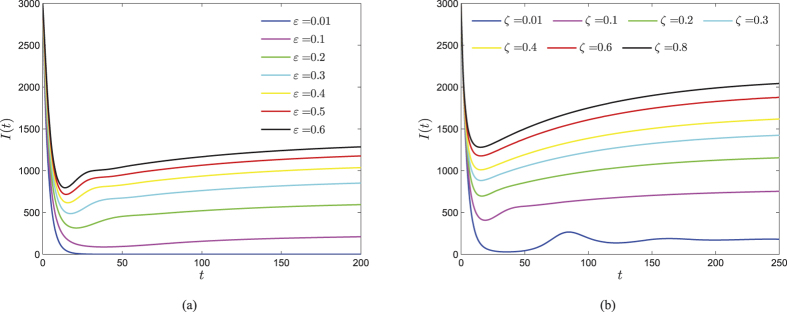
Consider system (1) with parameters *b* = 100, *d* = 0.01, *λ* = 0.0002, *η* = 0.3, *γ* = 0.3 and initial vector (1000, 1000, 3000, 2000). (**a**) Evolutions of the infected component of the case *ζ* = 0.1 with varying *ε*. (**b**) Evolutions of the infected component of the case *ε* = 0.25 with varying *ζ*.

Thus, Ψ is strictly increasing with respect to *ζ*. Besides, *ζ* is not incorporated in *R*_0_, and *ε* has positive effect on both Ψ and *R*_0_. Therefore, *I*^∗^ is strictly increasing with respect to *ζ* and *ε*, respectively. Figure 7(a,b) show how the parameters *ζ* and *ε* contribute web malware spread, respectively. The number of infected nodes undergoes a drastic change in the early time, and then would finally keep a balance. Higher values of *ζ* and *ε* will result a greater eventual level of malware-infected nodes, however, when both parameters *ζ* and *ε* reach great enough, the infected component of the malware equilibrium possesses less obvious increase.

**Figure 8:**
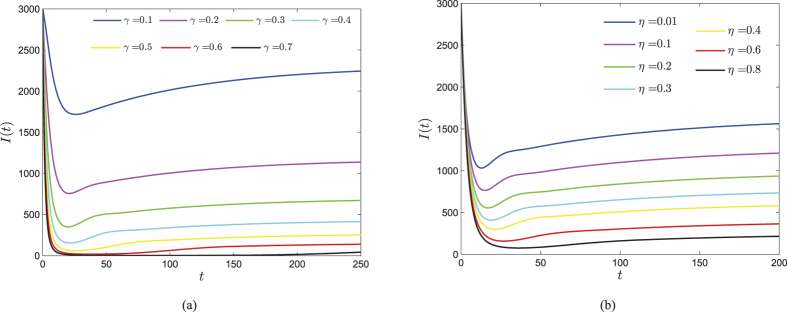
Consider system (1) with parameters *b* = 100, *d* = 0.01, *λ* = 0.0002, *ζ* = 0.1, *η* = 0.35, *ε* = 0.25, and initial vector (1000, 1000, 3000, 2000). (**a**) Evolutions of the infected component of the case *η* = 0.35 with varying *γ*. (**b**) Evolutions of the infected component of the case *γ* = 0.3 with varying *η*.

In contrast, the parameter *γ* has obvious negative effect on Ψ, and both *γ, η* have negative effects on *R*_0_. Furthermore, note that Ψ does not incorporate *η*, thus *I*^∗^ is strictly decreasing with respect to *γ* and *η*, respectively. Figure 8(a,b) show how the parameters *γ* and *η* inhibit the propagation of web malware, respectively. As *γ* and *η* increase, the level of infected nodes possesses less reduction, which indicates that the security investment is not proportional to the effectiveness of malware prevention. In other words, when the amount of security investment achieves a certain extent, user’s security awareness and the effects of anti-malware measures grow slowly.

### Optimal control and strategies

In system (1), there are four state variables *S(t*), *D(t*), *I(t*) and *R(t*). All the parameters in system (1) are constant, however, the real parameters should be time-varying. Thus, in this section, some of these parameters are considered to be controllable, and how to control the dynamic systems is worth studying^40^,^41^. We will use the control theory to obtain proper strategies for preventing malware spread over networks. First, we assume that the parameter *η* is controllable, and the variable function *η(t*) is introduced to reflect the probability that a D-node turns to be a R-node with the influence of user awareness at time *t*. Let 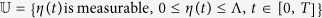 indicate an admissible control set.

The economic impact of malware attacks worldwide is dramatically increasing. As we all know, malware would cause massive direct damages and costs, such as labor costs, costs of repairing and cleansing infected systems, loss of user productivity, loss of revenue due to loss or degraded performance of system, and other costs directly incurred as the result of a malware attack. In order to effectively avoid malware attacks, updated anti-malware or firewall are widely deployed at both the organizational level and the individual level to defend against malware threats. But, these preventive measures also cost much security investment. Next, we aim to minimize the total cost of direct loss and security investment.

#### The optimal problem

The more nodes are infected by malware, the greater economic loss is. Thus, the financial loss caused by malware can be considered to be relevant to the number of infected nodes. We introduce a function *F*_*loss*_(*I(t*)) to describe the economic loss caused by the malware-infected nodes over the network. For simplicity, we suppose that the average loss caused by a single infected node per unit time is a suitable constant *ϕ*. Then, the whole loss caused by all the infected nodes within a unit time is *ϕI(t*) which is proportional to the infected node number. Then, we can compute the loss function across the time interval [0, *T*] as follows


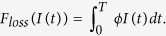


In addition, we also suppose that the level of user security awareness grows with the increasing of security investment. So, inversely, the cost investment function, denoted by *F*_*cost*_(*η(t*)), is also monotonically increasing with the value of *η(t*) which reflects the level of user security awareness. Here, we define


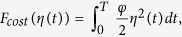


where *φ* is an appropriate coefficient. The greater *φ* is, the more security investment costs for same improving of user security awareness. The square of the control variable reflects the severity of the size effects of control.

In the sequel, we propose an optimal control problem to minimize the following objective functional


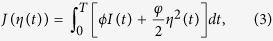


subject to


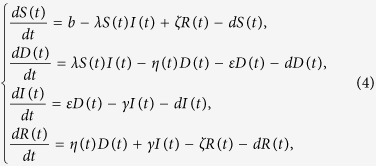


where *J(η*) is the sum of direct loss and preventive security investment.

For the sake of deriving an optimal solution pair, we need to define the Lagrangian and Hamiltonian for the optimal control problem (3) and (4). In fact, the Lagrangian of the optimal problem is given by


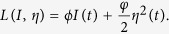


Next, we need to seek a suitable *η(t*) such that the integral of the above Lagrangian arrives the minimum. To do this, we define the Hamiltonian *H* for the control problem as follows


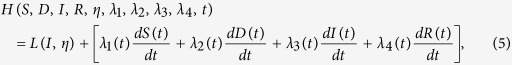


where *λ*_1_(*t*), *λ*_2_(*t*), *λ*_3_(*t*) and *λ*_4_(*t*) are the adjoint functions to be determined suitably.

**Theorem 1.** Consider system (4) with the objective functional (3), then there exists an optimal control 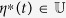 such that 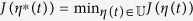.

Proof. Note that the control variable and the state variables in system (4) are nonnegative. Besides, the coefficients involved in system (4) are bounded and each state variable of system (4) is bounded on the finite time interval, so we can employ the result in ref. 42 (pp. 182) to confirm the existence of an optimal control to system (4).

First, the set of control and corresponding state variables is nonempty. All the right parts of the equations of system (4) are continuous, bounded and can be written as a linear function of *η* with coefficients depending on time and states. In this minimizing problem, the necessary convexity of the objective functional in *η(t*) is satisfied. The control space 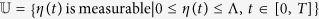 is apparently convex and closed. Besides, the optimal system is bounded which determines the compactness needed for the existence of the optimal control. Additionally, the integrand of the objective function (3), i.e., *I(t*) + *φη*^2^(*t*)/2, is convex on the control *η(t*). And, it is easy to confirm that there exists a constant *ρ* > 1 and positive numbers *v*_1_ and *v*_2_ such that *J(η(t*)) ≥ *v*_1_|*η*|^*ρ*/2^ + *v*_2_. Thus, we conclude that there exists an optimal control.

To find the optimal solution, the Pontryagin’s maximum principle is applied to show the existence of an optimal control.

**Theorem 2.** Let *S*^∗^(*t*), *D*^∗^(*t*), *I*^∗^(*t*) and *R*^∗^(*t*) be optimal state solutions associated with the optimal control variable *η*^∗^(*t*) for the optimal control problem. Then, there exist adjoint variables *λ*_1_(*t*), *λ*_2_(*t*), *λ*_3_(*t*) and *λ*_4_(*t*) that satisfy


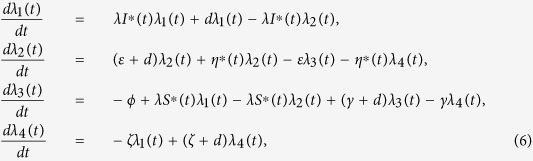


with transversality conditions


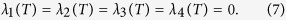


Furthermore, the optimal control *η*^∗^(*t*) is given by


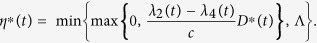


Proof. First, we use the Hamiltonian (5) to determine the adjoint equations and the transversality conditions. By setting *S(t*) = *S*^∗^(*t*), *D(t*) = *D*^∗^(*t*), *I(t*) = *I*^∗^(*t*) and *R(t*) = *R*^∗^(*t*), and differentiating the Hamiltonian (5) with respect to the state variables *S, D, I* and *R*, we obtain


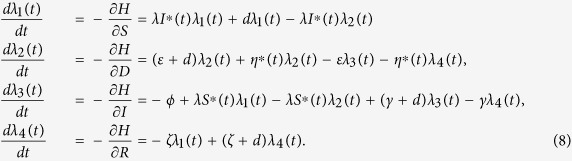


By the optimality conditions, we have


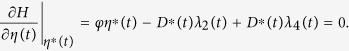


It follows by the above identity that


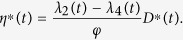


Considering the property of the control set 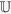, we obtain


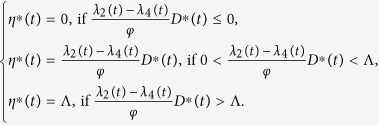


So we have the optimal control *η*^∗^(*t*) which can be written in the following compact notation


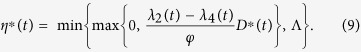


Here, the formula (9) for *η*^∗^ is called as the characterization of the optimal control. The optimal control and states can be found by solving the optimality system consisting of the state system (4) with boundary conditions, the adjoint system (6) and (7), and the characterization of the optimal control (9). To solve the optimality system, we use the initial and transversality conditions together with the characterization of the optimal control *η*^∗^ given by (9).

By substituting the values of *η*^∗^(*t*) into the control system (4), we get the following system


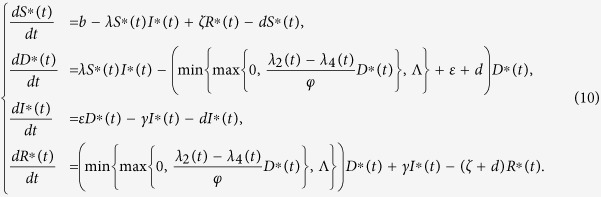


To find out the optimal control and the state system, we need to numerically solve the above system (10).

#### Numerical algorithm

In this section, we apply an iterative approach called *Gauss-Seidel-like implicit finite-difference method* to solve the optimality system. First, we discretize the time interval [0, *T*] into *n* sub-intervals at the points *t*_*k*_ = *kδ, k* = 0, 1, …, *n(nδ* = *T*), where *δ* is the time step. It is well known that the derivative of a differentiable function *x(t*) is defined by


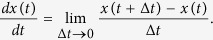


Thus, the time derivative of the state variable can be approximated by its first-order forward-difference when the time step *δ* is small enough, e.g.,


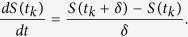


In the sequel, we denote *S*_*k*_ = *S(t*_*k*_), *D*_*k*_ = *D(t*_*k*_), *I*_*k*_ = *I(t*_*k*_), *R*_*k*_ = *R(t*_*k*_) and 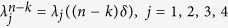. By the Gauss-Seidel-like implicit finite-difference method developed by Gumel *et al*.^43^, we can get


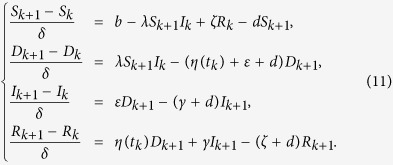


Then, the above state values can be used to solve the adjoint equations by approximating the time derivative of the adjoint variables using their first-order backward-differences because of the transversality conditions. Thus, we derive


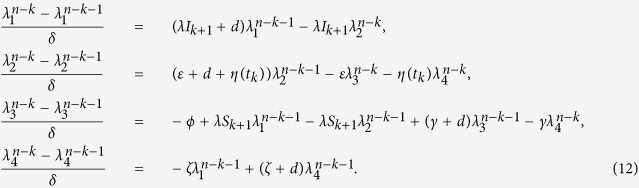


Next, we can formulate an algorithm to solve the optimality system and get the optimal control by certain calculations. It follows by (11) and (12) that


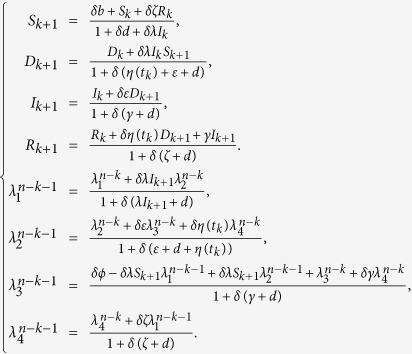


Then, by some calculations, it follows by (9) that the value of the optimal control at time *t*_*k*_ _+_ _1_ is formulated as


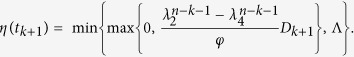


#### Numerical simulations

**Figure 9:**
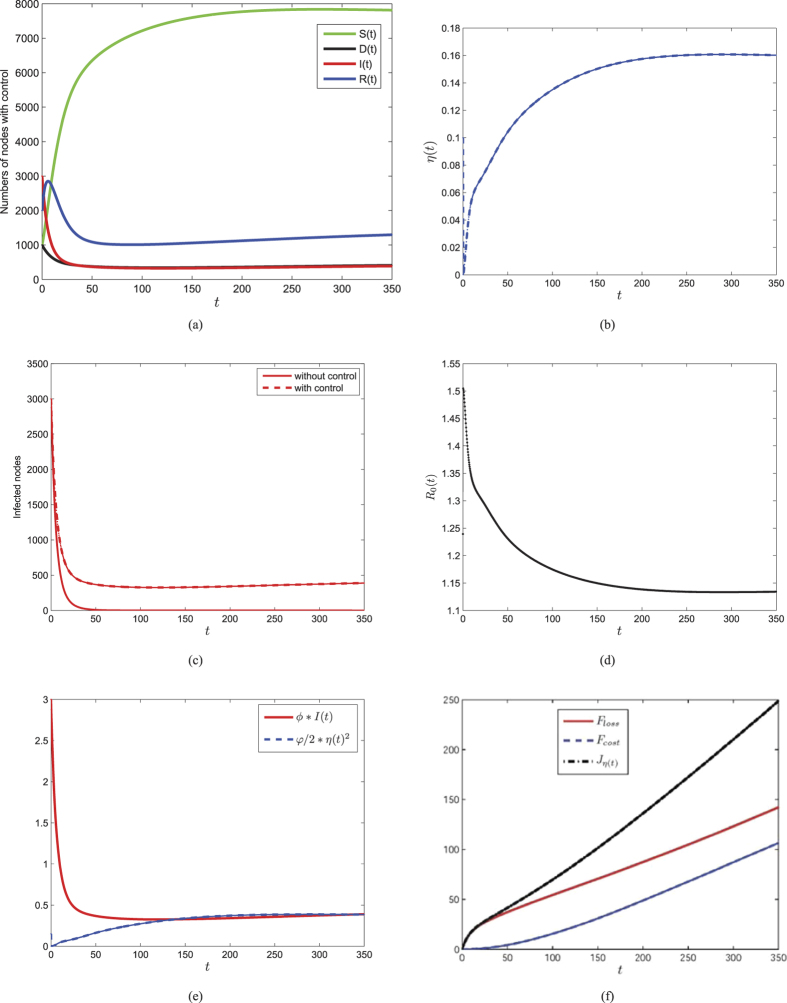
Plots of the control system (3) and (4) with parameters given above and with specific initial values *S*_0_ = 1000, *D*_0_ = 1000, *I*_0_ = 3000, *R*_0_ = 2000. (**a**) Optimal solutions of the state variables. (**b**) Plot of the optimal control *η(t*). (**c**) Comparison of infected nodes *I(t*) with control and without control. (**d**) Plot of the threshold *R*_0_(*t*) with varying *η(t*). (**e**) Plot of integrands in loss and cost functions. f) Plot of the objective function and the loss and cost functions.

In this section, we aim to do some numerical simulations for the optimality system by using the above iterative method. In order to compare the numerical results of system (1) and the control system, we consider the same parameters, i.e., *b* = 100, *d* = 0.01, *λ* = 0.00005, *ζ* = 0.1, *η* = 0.5, *ε* = 0.2, *γ* = 0.2, and *ϕ* = 0.001, *φ* = 30. Through certain calculations, we plot Fig. 9(a) that shows the evolutions of the numbers of nodes in each compartment with the optimal control shown in Fig. 9(b). We can see in Fig. 9(b) that the optimal control *η(t*) increases in the early time and finally tends to a constant. This means that we should enlarge the security investment in the process of control. Figure 9(c) illustrates the evolution of infected nodes with optimal control, compared to the number of infected nodes without control.

**Figure 10:**
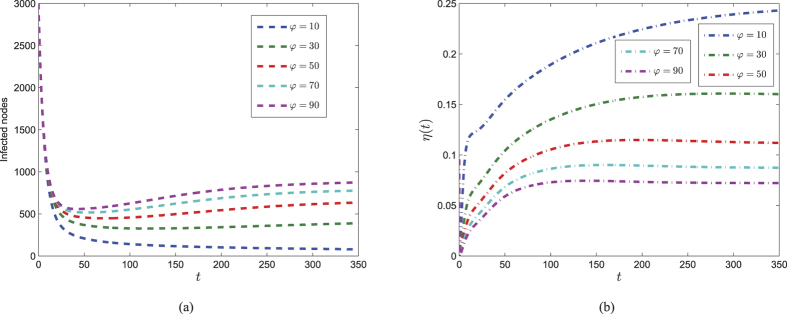
The impact of *φ* on dynamics of optimal control system (3) and (4). (**a**) Evolutions of infected numbers with respect to varying *φ*. (**b**) Trajectories of optimal control of *η(t*) with respect to five different values of *φ*.

In order to explore the influence of parameter *φ*, we design a numerical experiment with *φ* as a variable. Consider other parameters given above, Fig. 10 shows the dynamics of system (3) and (4) with five different values of *φ*. It is shown in Fig. 10(b) that the control variable decreases and approaches the equilibrium earlier with the increase of *φ*, while Fig. 10(a) shows that the number of infected nodes increases for greater value of *φ*. This indicates that security investment should be properly cut down when the cost arrives high enough.

## Discussion

In this study, we introduce several parameters to describe the spread processes of web malware based on their mechanism analysis, and develop a new compartmental SDIRS model with varying network size to model the spread of web malware over networks. We compute the propagation threshold of the model and carry out its sensitivity analysis. The properties of the elementary model system are also carefully analyzed. If the threshold is below unity, the global stability of the malware-free equilibrium is theoretically proved. The malware equilibrium is proved to be locally stable if the threshold exceeds unity. Although we study the long behavior of this model, it can be only used to describe web malware spread within a short time interval since the parameters in the SDIRS model are assumed to be constant.

Practical parameters are actually varying with time. So, based on the newly established SDIRS model, we consider the parameter *η* to be varying and controllable. Aiming to keep the total economic loss of security investment and infection loss as low as possible, we propose an objective functional and study the optimal control strategy towards the *η* parameter. Through theoretical analysis and the Pontryagin’s maximum principle, the expression of the optimal control is explicitly given. Numerical simulations show the effectiveness of taking the control strategy on inhibiting the spread of web malware over networks. Also, we suggest that users should enhance their awareness levels of network security, such as being able to discriminate malicious links and not to click on strange hyperlinks, installing updated anti-virus software on devices, keeping browsers updated and installing patches immediately.

We develop the model (1) based on a homogeneously mixed assumption of the propagation network. It can be applied to model the proliferation of web malware over complete or regular networks. But, most real-world networks, such as the WWW and Internet, have been empirically found to be highly structured rather than simply homogeneously, e.g., each device may have heterogeneous malicious hyperlinks. The compartment-based models suffer from a common defect of not making full use of the knowledge concerning the structure of the propagation network. As a result, it is worth understanding the impact of network topology on the web malware prevalence. In recent years, network(node)-based models have already been considered and developed to model infectious disease diffusion over complex networks^44^,^45^, such as spatial epidemics^46^ and waterborne diseases^47^. Thus, our future work is to formulate further novel network-based models by incorporating the influence of network topology on web malware spread.

## Methods

### Calculation of the threshold

Van den Driessche and Watmough^48^ developed a standard approach for calculating the spread threshold of compartmental models. For convenience, we first introduce it here.

We consider an *n*–dimensional deterministic system for modeling virus propagation, where the first *m* variables correspond to all infected compartments which are numbered as compartment 1 through *m*, and the left *n* − *m* compartments which are numbered as compartment *m* + 1 through *n* correspond to uninfected nodes. Denote a variable vector **x** = (*x*_1_, *x*_2_, …, *x*_*n*_), where *x*_*i*_ denote the number (or proportion) of nodes in the *i*-th compartment. Let 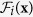 be the rate of appearance of new infections into compartment *i*, 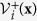 be the rate of transfer of nodes into compartment *i* by all other means, and 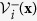 be the rate of transfer of nodes out of compartment *i*. Then the considered model can be shown as follows


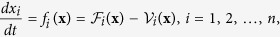


where 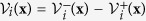. Let 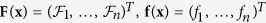, and 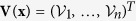. For the functions in the above system, five assumptions (A1)–(A5) are described below.

- If **x** ≥ 0, then 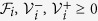 for *i* = 1, 2, …, *n*.

- If *x*_*i*_ = 0, then 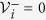. In particular, if **x** ∈ *X*_*s*_: = {**x** ≥ 0|*x*_*i*_ = 0, *i* = 1, …, *m*}, which is defined as the set of all infection-free states, then 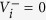 for *i* = 1, …, *m*.

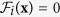
 if *i* > *m*.

- If **x** ∈ *X*_*s*_, then 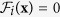 and 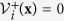 for *i* = 1, …, *m*.

- If 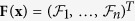 is set to zero, then all eigenvalues of *D***f**(**x**_0_) have negative real parts, where *D***f**(**x**_0_) is the derivative [∂*f*_*i*_/∂*x*_*i*_](i.e., Jacobian matrix) evaluated at **x**_0_ which is a (locally asymptotically) stable equilibrium.

Then, van den Driessche and Watmough proved a useful lemma (see Lemma 1 in the ref. 48). That is, if the above assumptions (A1)–(A5) are satisfied, then the derivatives *D***F**(**x**_0_) and *D***V**(**x**_0_) are partitioned as


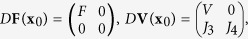


where *F* and *V* are the *m* × *m* matrices defined by


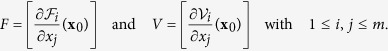


Further, *F* is non-negative, *V* is a non-singular matrix and all eigenvalues of *J*_4_ have positive real part. Then, the threshold can be defined as


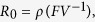


where *ρ(A*) denotes the spectral radius of a matrix *A* (refer to the ref. 48).

Next, in order to compute the threshold of the compartmental model, we denote


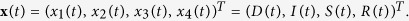


Then the SDIRS model can be rewritten as follows


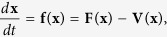


where 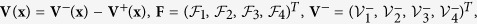 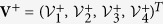, and


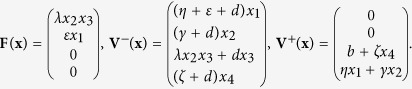


It is easy to verify that the functions satisfy assumptions (A1)–(A5).

- If **x** ≥ 0, then 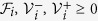 for *i* = 1, 2, 3, 4.

- If *x*_*i*_ = 0, then 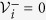. In particular, if *x* ∈ *X*_*s*_, then 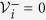 for *i* = 1, 2.

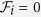
 if *i* > 2.

- If **x** ∈ *X*_*s*_, then 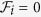 and 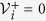 for *i* = 1, 2.

- If **F**(**x**) is set to zero, then all eigenvalues of *D***f**(**x**_0_) have negative real parts.

Then, it follows by the above result (see also Lemma 1 in the ref. 48) that 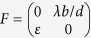, 
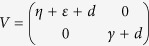
. Then, the threshold is the spectral radius of the matrix *FV*^−1^, i.e.,


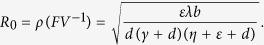


### Proof of the global stability of malware-free equilibrium 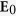


**Theorem 3.** The malware-free equilibrium point 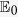 of model system (1) is globally asymptotically stable with respect to Ω if *R*_0_ < 1.

Proof. We proceed by use of the Lyapunov direct method with undetermined coefficients. Denote 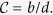 Consider the following candidate function


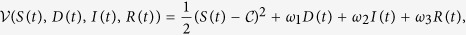


where *ω*_1_, *ω*_2_, *ω*_3_ are positive constants to be determined. Clearly, it follows by *D(t*) ≥ 0, *I(t*) ≥ 0 and *R(t*) ≥ 0 that 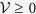. Furthermore, we have 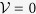 if and only if 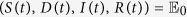 with respect to Ω. That is, 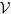 is positive definite.

The time derivative of 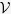 along an orbit of system (1) is


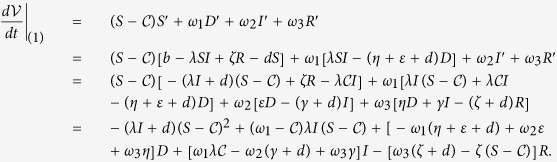


Let 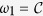, then we need to find appropriate *ω*_2_ such that the following two inequalities are satisfied


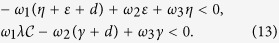


Define 
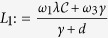
 and 
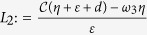
. Obviously, *L*_2_ is positive provided 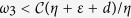.

It follows by (13) that *L*_1_ < *ω*_2_ < *L*_2_. Thus, *L*_2_ > *L*_1_ is necessary for the existence of *ω*_2_. Note that


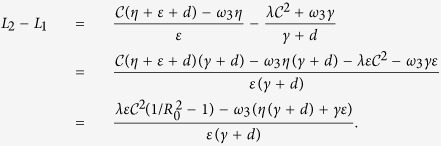


It follows by *R*_0_ < 1 that *L*_2_ − *L*_1_ > 0 provided


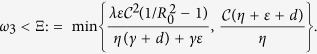


We know that *N(t*) = *b/d* + *ce*^−*dt*^, which is monotone and 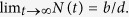 *N*(0) = *b/d* + *c* represents the initial size of the network. Next, we proceed by considering two cases.

**Case 1**: *c* < 0. In this case, *N*(0) < *b/d* and *N(t*) is strictly increasing. That is, the network size keeps growing to the maximum limit of *b/d*. Thus, we have 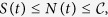 which implies that 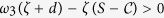 provided *ω*_3_ is positive. Thus, 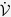 is negative definite inside the region of 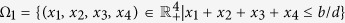 provided 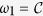, *ω*_2_ ∈ (*L*_1_, *L*_2_) and *ω*_3_ < Ξ. Therefore, 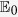 is globally asymptotically stable with respect to Ω_1_.

**Case 2**: *c* > 0. In this case, *N*(0) > *b/d* and *N(t*) is strictly decreasing. That is, the network size keeps reducing to the minimum limit of *b/d*. Thus, we have 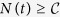 and *S(t*) ≤ *N(t*) ≤ *N*(0).

Let 
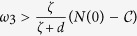
, then


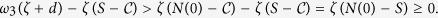


Since *N(t*) is strictly decreasing with 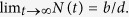 Without loss of generality, we assume that


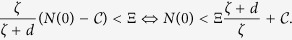


Let 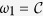, *ω*_2_ ∈ (*L*_1_, *L*_2_) and 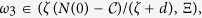 then


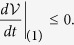


Furthermore, it is easily verified that 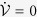 if and only if 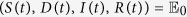 with respect to 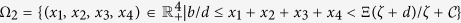. That is, 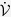 is negative definite. Therefore, 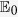 is globally asymptotically stable with respect to Ω_2_.

The proof completes by following the above two cases.

### Proof of the stability of malware equilibrium 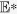


**Theorem 4.** The malware equilibrium 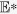 of system (1) is asymptotically stable if *R*_0_ > 1.

Proof. Here, we use the method of first approximation to show the asymptotic stability of 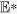. By certain calculations, the Jacobian matrix of (1) at a point 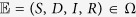 can be derived as


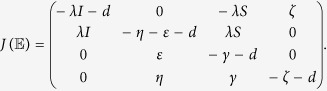


Thus, the Jacobian matrix of (1) at the malware equilibrium 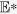 is


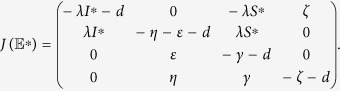


Next, we only need to confirm the matrix 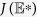 is stable, namely, the real parts of all its eigenvalues are negative. This is usually done by checking the Routh-Hurwitz conditions, but here verification of the inequalities in the Routh-Hurwitz conditions for 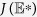 is technically rather complicated. So, we use another criteria for the stability of matrices. That is, for an *m* × *m* matrix *A* with real entries to be stable, it is necessary and sufficient that: (1) the second *compound matrix* (See *Methods D) A*^[2]^ is stable; (2) 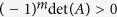. This result was developed by Li *et al*.^39^ by using the spectral properties of the second compound matrices (also see Lemma 5.1 in ref. 39).

Thus, it remains to show that 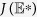 satisfies the above conditions (1) and (2). The second compound matrix 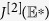 of the Jacobian matrix 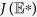 is


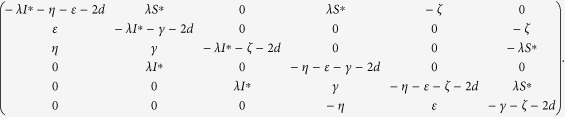


For the diagonal matrix *P* = diag (*I*^∗^, (*γ* + *d*)/(*ε)I*^∗^, *t*_1_*I*^∗^, *S*^∗^, *t*_2_*S*^∗^, *t*_3_*S*^∗^), where *t*_1_, *t*_2_, *t*_3_ are positive real constants to be determined, then the matrix 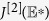 is similar to 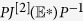



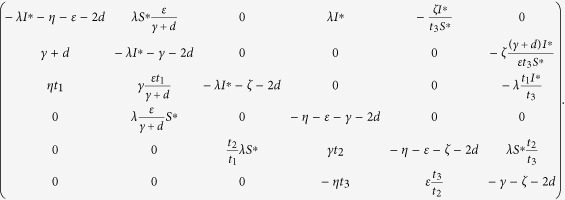


The matrix 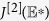 is stable if and only if 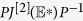 is stable, for similarity preserves the eigenvalues. Since the diagonal elements of the matrix 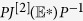 are negative, an easy argument applying Geršgorin discs shows that it is stable if it is diagonally dominant in rows. Denote 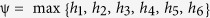, where


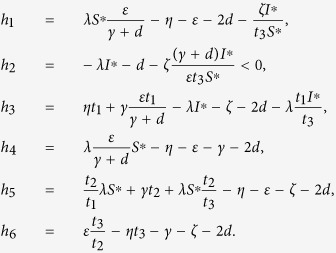


It follows by the expression of the threshold *R*_0_ and 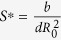 that


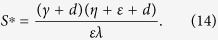


Substituting (14) into *h*_1_ and *h*_4_ yields *h*_1_ = −*d* − (*ζI*^∗^)/(*t*_3_*S*^∗^) < 0 and *h*_4_ = −*γ* − *d* < 0.

By choosing *t*_1_ = (*γ* + *d*)(*ζ* + 2*d*)/(*η(γ* + *d*) + *εγ*), then we have


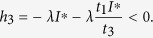


Since *t*_1_ is already fixed, it is sufficient to determine the values of *t*_2_, *t*_3_ such that both *h*_5_ and *h*_6_ are negative. We assume that *t*_2_ < (*d* + *ζ*)/*γ* and set


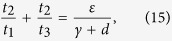


then


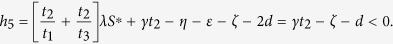


It follows by (15) that *t*_2_ is monotonically increasing as a function of *t*_3_. If *t*_3_ → 0, then *t*_2_ → 0, *t*_2_/*t*_3_ → (*ε*)/(*γ* + *d*), and


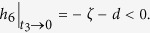


Thus, we can always choose a proper *t*_3_ > 0 small enough such that *h*_6_ < 0 and *t*_2_ given by (15) is less than (*d* + *ζ*)/*γ*. Therefore, we have *ψ* < 0, which implies the diagonal dominance as claimed and thus verifies the above condition (1).

The determinant of 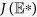 can be computed as


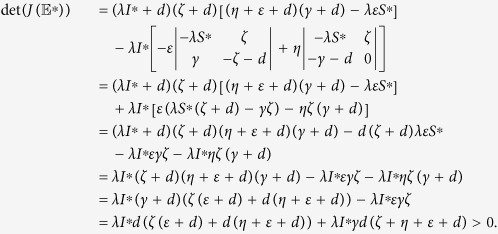


This verifies the above condition (2) and completes the proof.

**Remark**: If *ζ* = 0, then system (1) can be also reduced to model the case that recovered nodes have permanent immunity. Let *s(t*) = *S(t*)/*N(t*), *d(t*) = *D(t*)/*N(t*), *i(t*) = *D(t*)/*N(t*) and *r(t*) = *D(t*)/*N(t*) denote the fractions of the compartments *S, D, I, R* in the population, respectively. Then system (1) becomes


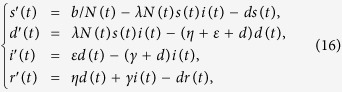


subject to the restriction *s(t*) + *d(t*) + *i(t*) + *r(t*) = 1. In system (16), the term *b/N(t*) represents the percentage of newly-connected S-nodes over the whole network within unit time, *λN(t*) means the average infection rate of I-nodes over the whole network per unit time. Next, we denote 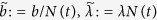, and assume that 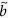, 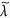 keep constant here, which indicates that in this case *b* and *λ* are actually changing with the varying network size.

Observe that the variable *r(t*) does not appear in the first three equations of (16) and note that the identity *s(t*) + *d(t*) + *i(t*) + *r(t*) = 1 implies 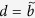. This allows us to attack (16) by studying the subsystem


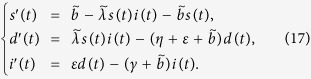


From physical considerations, we study (16) in the closed set 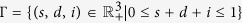. It can be verified that Γ is positively invariant with respect to (17). We denote by ∂Γ and Γ the boundary and the interior of Γ, respectively. Note that system (17) is essentially equivalent to a special case (*α* = 0) of system (2.3) in ref. 39. Thus, we can similarly address the global stability of the malware-free equilibrium (respectively, malware equilibrium) of system (17) with respect to Γ (respectively, Γ) by the method given in ref. 39.

### Compound matrices

For an *n* × *n* matrix *A* and integer 1 ≤ *k* ≤ *n*, the *k*-th additive compound matrix of *A* is denoted by *A*^[*k*]^. This is an *N* × *N* matrix, 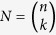, defined by


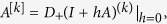


where *B(k*) is the *k*th exterior power of an *n* × *n* matrix *B* and *D*_+_ denotes the right-hand derivative. Some details for the definition and properties of additive compound matrices together with their connections to differential equations can be referred to the papers^49^,^50^.

The entries in *A*^[2]^ are linear relations of those in *A*. Let *A* = (*a*_*ij*_). For any integer 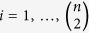, let (*i*) = (*i*_1_, *i*_2_) be the *i*th member in the lexicographic ordering of integer pairs such that 1 ≤ *i*_1_ < *i*_2_ ≤ *n*. Then, the entry in the *i*th row and the *j*th column of *Z* = *A*^[2]^ is defined by


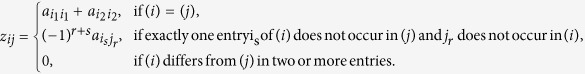


Pertinent to our purpose, for *n* = 4, the second additive compound matrix *A*^[2]^ of an *n* × *n* matrix *A* = (*a*_*ij*_) is


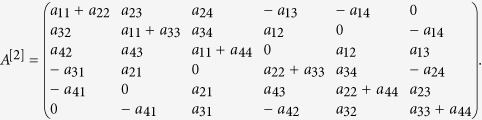


For any integer 1 ≤ *k* ≤ *n*, the *k*th additive compound matrix *A*^[*k*]^ of *A* is defined canonically. Some properties of the additive compound matrices and further applications can be found in the refs 50 and 51.

## Additional Information

**How to cite this article**: Liu, W. and Zhong, S. Web malware spread modelling and optimal control strategies. *Sci. Rep.* **7**, 42308; doi: 10.1038/srep42308 (2017).

**Publisher's note:** Springer Nature remains neutral with regard to jurisdictional claims in published maps and institutional affiliations.

## References

[b1] Anthe C. . Microsoft Security Intelligence Report Volume 20 (July–December 2015). http://www.microsoft.com/security/sir/default.aspx (2015) (Date of access: 10th September, 2016).

[b2] Weinberger S. Computer security: Is this the start of cyberwarfare? Nature 474, 142–145 (2011). 21654779 10.1038/474142a

[b3] Internet Live Stats. http://www.internetlivestats.com/ (2016) (Date of access: 25th September, 2016).

[b4] Sellke S. H., Shroff N. B. & Bagchi S. Modeling and automated containment of worms. IEEE T. Depend. Secure 5(2), 71–86 (2008).

[b5] Liu W., Liu C. & Liu X. A discrete dynamic model for computer worm propagation. Springer Proceedings in Mathematics & Statistics, 150, 119–131 (2015).

[b6] Song L., Jin Z., Sun G., Zhang J. & Han X. Influence of removable devices on computer worms: Dynamic analysis and control strategies. Compu. Math. Appl. 61, 1823–1829 (2011).

[b7] Castellano C., Fortunato S. & Fortunato S. Statistical physics of social dynamics. Rev. Mod. Phys. 81, 0034 (2009).

[b8] Hu H. . WiFi networks and malware epidemiology. Proc. Nat. Acad. Sci. 106, 1318 (2009). 19171909 10.1073/pnas.0811973106 PMC2635807

[b9] Marchal S., FranÇois J., State R. & Engel T. PhishStorm: detecting phishing with streaming analytics. IEEE Trans. Netw. Service Manag. 11(4), 458–471 (2014).

[b10] Li L. Patch invasion in a spatial epidemic model. Appl. Math. Comput. 258, 342–349 (2015)

[b11] Sun G.-Q. & Zhang Z.-K. Global stability for a sheep brucellosis model with immigration. Appl. Math. Comput. 246, 336–345 (2014).

[b12] Li M.-T., Sun G.-Q., Wu Y.-F., Zhang J. & Jin Z. Transmission dynamics of a multi-group brucellosis model with mixed cross infection in public farm. Appl. Math. Comput. 237, 582–594 (2014).

[b13] Sun G.-Q., Wu Z.-Y., Wang Z. & Jin Z. Influence of isolation degree of spatial patterns on persistence of populations. Nonlinear Dyn. 83, 811–819 (2016).

[b14] Sun G.-Q. Mathematical modeling of population dynamics with Allee effect. Nonlinear Dyn. 85, 1–12 (2016).

[b15] Li L. & Jin Z. Pattern dynamics of a spatial predator–prey model with noise. Nonlinear Dyn. 67, 1737–1744 (2012).

[b16] Sun G.-Q., Zhang J., Song L.-P., Jin Z. & Li B.-L. Pattern formation of a spatial predator–prey system. Appl. Math. Comput. 218, 11151–11162 (2012)

[b17] Li L., Jin Z. & Li J. Periodic solutions in a herbivore-plant system with time delay and spatial diffusion. Appl. Math. Model. 40, 4765–4777 (2016).

[b18] Sun G.-Q., Wang S.-L., Ren Q., Jin Z. & Wu Y.-P. Effects of time delay and space on herbivore dynamics: linking inducible defenses of plants to herbivore outbreak. Sci. Rep. 5, 11246 (2015) 26084812 10.1038/srep11246 PMC4471659

[b19] Sun G.-Q. . Influence of time delay and nonlinear diffusion on herbivore outbreak. Commun. Nonlinear Sci. Numer. Simulat. 19, 1507–1518 (2014).

[b20] Sun G.-Q. Pattern formation of an epidemic model with diffusion. Nonlinear Dyn. 69, 1097–1104 (2012). 10.1007/s11071-012-0330-5 PMC7088525 32214667

[b21] Liu W., Liu C., Liu X., Cui S. & Huang X. Modeling the spread of malware with the influence of heterogeneous immunization. Appl. Math. Model. 40(4), 3141–3152 (2016).

[b22] Carter K. M., Idika N. & Streilein W. W. Probabilistic threat propagation for network security. IEEE T. Inf. Foren. Sec. 9(9), 1394–1405 (2014).

[b23] Gil S., Kott A. & Barabási A.-L. A genetic epidemiology approach to cyber-security. Sci. Rep. 4, 5659 (2014). 25028059 10.1038/srep05659 PMC4100021

[b24] Misra A. K., Verma M. & Sharma A. Capturing the interplay between malware and anti-malware in a computer network. Appl. Math. Comput. 229, 340–349 (2014).

[b25] Liu W., Liu C., Yang Z., Liu X., Zhang Y. & Wei Z. Modeling the propagation of mobile malware on complex networks. Commun. Nonlinear Sci. 37, 249–264 (2016).

[b26] Li C., van de Bovenkamp R. & van Mieghem P. Susceptible-infected-susceptible model: A comparison of N-intertwined and heterogeneous mean-field approximations. Phys. Rev. E 86, 026116 (2012). 10.1103/PhysRevE.86.026116 23005834

[b27] Parshani R., Carmi S. & Havlin S. Epidemic Threshold for the Susceptible-Infectious-Susceptible Model on Random Networks. Phys. Rev. Lett. 104(25), 258701 (2010). 20867419 10.1103/PhysRevLett.104.258701

[b28] Diekmann O., Heesterbeek J. A. P. & Metz J. A. J. On the definition and the computation of the basic reproduction ratio R0 in models for infectious diseases in heterogeneous populations. J. Math. Biol. 28, 365–382 (1990). 2117040 10.1007/BF00178324

[b29] Wang W. Predicting the epidemic threshold of the susceptible-infected-recovered model. Sci. Rep. 6, 24676 (2016). 27091705 10.1038/srep24676 PMC4835734

[b30] Mishra B. K., Haldar K. & Sinha D. N. Impact of information based classification on network epidemics. Sci. Rep. 6, 28289 (2016). 27329348 10.1038/srep28289 PMC4916446

[b31] Kitsak M. . Identification of influential spreaders in complex networks. Nat. Phys. 6, 888 (2010).

[b32] Iframe virus. https://en.wikipedia.org/wiki/Iframe_virus (2016) (Date of access: 29th September, 2016).

[b33] Holz T., Marechal S. & Raynal F. New threats and attacks on the World Wide Web. IEEE Security & Privacy, 4(2), 72–75 (2006).

[b34] Wu L., Du X. & Wu J. Effective defense schemes for phishing attacks on mobile computing platforms. IEEE T. Veh. Technol. 65(8), 6678–6691 (2016).

[b35] Li M. Y., Smith H. L. & Wang L. Global dynamics of an SEIR epidemic model with vertical transmission. SIAM J. Appl. Math. 62, 58–69 (2001).

[b36] Butler G. J. & Waltman P. Persistence in dynamical systems. Proc. Am. Math. Soc. 96, 425 (1986).

[b37] Thieme H. Epidemic and demographic interaction in the spread of potentially fatal diseases in growing populations, Math. BioSci. 111, 99 (1992). 1515742 10.1016/0025-5564(92)90081-7

[b38] Freedman H. I., Tang M. X. & Ruan S. G. Uniform persistence and flows near a closed positively invariant set, J. Dynam. Diff. Equat. 6, 583 (1994).

[b39] Li M. Y., Graef J. R., Wang L. & Karsai J. Global dynamics of a SEIR model with varying total population size. Math. Biosci. 160, 191–213 (1999). 10.1016/s0025-5564(99)00030-9 10472754

[b40] Li H., Chen G., Huang T. & Dong Z. High-performance consensus control in networked systems with limited bandwidth communication and time-varying directed topologies. IEEE T. Neur. Net. Lear. Accepted in press. doi: 10.1109/TNNLS.2016.2519894. 26887015

[b41] Zhang C., Zhou S., Miller J. C., Cox I. J. & Chain B. M. Optimizing hybrid spreading in metapopulations. Sci. Rep. 5, 9924 (2015). 25923411 10.1038/srep09924 PMC4413882

[b42] Lukes D. L. Differential equations: classical to controlled. In: Mathematics in Science and Engineering, Academic Press, New York, 162, 182 (1982).

[b43] Gumel A. B., Shivakumar P. N. & Sahai B. M. A mathematical model for the dynamics of HIV-1 during the typical course of infection. Third World Congress of Nonlinear Analysts 47, 2073–2083 (2001).

[b44] Wang Y., Cao J., Alofi A., AL-Mazrooei A. & Elaiw A. Revisiting node-based SIR models in complex networks with degree correlations. Physica A 437, 75–88 (2015).

[b45] Wang Y. . Global analysis of an SIS model with an infective vector on complex networks. Nonlinear Anal-Real 13(2), 543–557 (2012).

[b46] Sun G.-Q., Jusup M., Jin Z., Wang Y. & Wang Z. Pattern transitions in spatial epidemics: Mechanisms and emergent properties. Phys. Life Rev. http://dx.doi.org/10.1016/j.plrev.2016.08.002 (2016). 10.1016/j.plrev.2016.08.002 PMC7105263 27567502

[b47] Wang Y. & Cao J. Global dynamics of a network epidemic model for waterborne diseases spread. Appl. Math. Comput. 237, 474–488 (2014).

[b48] Van den Driessche P. & Watmough J. Reproduction numbers and sub-threshold endemic equilibria for compartmental models of disease transmission. Math. BioSci. 180, 29–48 (2002). 12387915 10.1016/s0025-5564(02)00108-6

[b49] London D. On derivations arising in differential equations. Linear Multilinear A. 4, 179–189 (1976).

[b50] Muldowney J. S. Compound matrices and ordinary differential equations. Rocky Mountain J. Math. 20, 857–872 (1990).

[b51] Li Y. & Muldowney J. S. On Bendixson’s criterion. J. Differential Equations 106, 27–39 (1993).

